# OA04.04. Changes in physiological and psychological markers of stress in hospital personnel after a low-dose mindfulness-based worksite intervention

**DOI:** 10.1186/1472-6882-12-S1-O16

**Published:** 2012-06-12

**Authors:** M Klatt, B Steinberg, D Marks, A Duchemin

**Affiliations:** 1 The Ohio State University College of Medicine, Columbus, USA

## Purpose

To determine the efficacy of a pragmatic low dose mindfulness-based worksite intervention on biological and behavioral indices of stress in healthcare professionals caring for seriously ill patients.

## Methods

Participants (n=32) were recruited among the Surgical Intensive Care Unit (SICU) personnel of a large university hospital, and were randomized to intervention or wait-list control groups, stratified by gender. The low dose 8-week mindfulness-based intervention was delivered on site, one hour before shift change.

## Results

Participants were representative of the SICU staff with 69% nurses, 88% females, age average of 44, and 11.8 (±10.1, SD) average years working in the SICU. Participant biological indices of stress, measured by the level of salivary α-amylase, was significantly reduced in the intervention group (t=2.562, p=0.026) only. Behaviorally, they rated their experience of stress using the Depression, Anxiety and Stress Scale (DASS-21), and rated sleep over the past month using the Pittsburg Sleep Quality Index (PSQI). There was a significant decrease of the scores on the DASS-21 stress subscale (t=2.245, p=0.040) and a significant improvement in the overall quality of sleep (t=2.482, p=0.027) between pre and post assessments in the intervention group with no changes for the wait list group. Work satisfaction also increased significantly (t=-3.2020, p=0.006) for the intervention group only.

## Conclusion

These data indicate the effectiveness of a mindfulness-based intervention delivered at the worksite towards stress reduction for staff in a high stress, hospital environment. The SICU personnel care for trauma 1 and 2 level patients and patients with severe pathology recovering from major surgery, and are confronted with catastrophic events on a regular basis. Given the nature of the job, work-related stressful events in the SICU will not change, but the resiliency tools offered via the intervention may help maintain wellness and prevent the deleterious effects of stress.

